# [Corrigendum] Tanshinone I effectively induces apoptosis in estrogen receptor-positive (MCF-7) and estrogen receptor-negative (MDA-MB-231) breast cancer cells

**DOI:** 10.3892/ijo.2026.5928

**Published:** 2026-08-12

**Authors:** Irina Tsoy Nizamutdinova, Gyeong Won Lee, Kun Ho Son, Su Jin Jeon, Sam Sik Kang, Yeong Shik Kim, Jae Heun Lee, Han Geuk Seo, Ki Churl Chang, Hye Jung Kim

Int J Oncol 33: 485-491, 2008; DOI: 10.3892/ijo_00000031

Subsequently to the publication of the above article, an interested reader drew to the authors' attention that it was unexpected that the authors would have identified an increase in the levels of cleaved caspase-3 in the MCF-7 cell line in this study, as reported in the Results section on p. 487, four lines from the bottom of the right-hand column, since it has been shown that MCF-7 cells do not express caspase-3 (for example, see the paper by Reiner U. Jänicke and colleagues entitled 'MCF-7 breast carcinoma cells do not express caspase-3'. Breast Cancer Res. Treat. vol. 117.1 (2009); p. 219-221).

In their response, the authors provided details of the source and authentication status of the MCF-7 cell line used in this study. The MCF-7 cells were purchased from American Type Culture Collection (ATCC #HTB-22, Manassas, VA) and maintained for fewer than 20 passages. Cells were maintained in RPMI 1640 medium supplemented with 10% fetal bovine serum, 1% penicillin and streptomycin, and incubated at 37°C in a humidified atmosphere containing 5% CO_2_. The authors also provided the STR profiling details to confirm the authenticity of the MCF-7 cells used in this study.

In addition, concerning the results of the experiments showing that Tanshinone I actives the caspase-3 pathway in the MCF-7 breast cancer cells (shown in the western blots portrayed in Fig. 3A), the authors explained that original uncropped TIFF files are no longer available due to laboratory data retention policies from that era; however, other studies in the research literature have reported identical caspase-3-like immunoreactivity patterns in MCF-7 cells using the same antibodies, reflecting caspase-7 cross-reactivity (for example, nine precedent papers are included in the references of the paper by Jänicke and colleagues mentioned by the reader above).

Therefore, the authors consider that the reader's concern would be adequately addressed by referring to 'effector caspase activation' in place of caspase-3 activation, with an explicit acknowledgement made of the reported MCF-7 caspase-3 deficiency in the revised text in the paper, also including three additional reference citations. This conservative re-interpretation of the data in this paper should eliminate any interpretive concerns.

Therefore, the authors wish to propose the following textual revisions in the published paper:

- In the Abstract, 20 lines down, 'caspase 3' should have read as '**effector caspase**';
- In the key words, 'caspase 3' should be replaced with '**caspase**';
- The second subheading in the Results section (p. 487, the right-hand column) should be changed to read as '*Tanshinone I induces apoptotic cell death by decreasing the Bcl-2/Bax ratio and activating* ***effector caspases*** *in breast cancer cells*'.
- In this subsection, 14 lines down, the final sentences in this section should be changed to read as follows: **'Concurrently, tanshinone I dose-dependently increased signal intensity detected by anti-cleaved caspase-3 antibody in both breast cancer cell lines (Fig. 3A and B). Importantly, MCF-7 cells lack functional caspase-3 protein due to a 47-bp deletion in CASP3 exon 3 that abrogates translation (23a-23c). Thus, this immunoreactivity in MCF-7 may reflect cross-reactivity with activated caspase-7 or other effector caspases compensating for caspase-3 deficiency**. Interestingly, similar to Fig. 2, low dose tanshinone I (1 or 5 μM) more effectively induced **effector caspase immunoreactivity** in MDA-MB-231 cells. These results **demonstrate** that tanshinone I induces apoptotic cell death in breast cancer cells through the downregulation of the Bcl-2/Bax ratio **and activation of compensatory effector caspase pathways, even in caspase-3-deficient MCF-7 cells'.**
- The following three references (refs. 23a-23c), as cited in the revised text described in iv) above, should be included in the reference list:
  **23a. Janicke RU, Sprengart ML, Wati MR, Porter AG: Caspase3 is required for DNA fragmentation and morphological changes associated with apoptosis. J Biol Chem 273: 9357–9360, 1998.**
  **23b. Janicke RU: MCF-7 breast carcinoma cells do not express caspase-3. Breast Cancer Res Treat 117: 219-221. 2009.**
  **23c. Wang S, He M, Li L, Liang Z, Zou Z, Tao A: Cell-in-Cell Death Is Not Restricted by Caspase-3 Deficiency in MCF-7 Cells. J Breast Cancer 19: 231-241, 2016**
- In the Discussion, p. 490, left-hand column, second paragraph, 20 lines down, the three sentences that commence here should be changed to read as follows; 'In concert with these changes, **effector caspase activation** was induced by tanshinone I in a dose-dependent manner (Fig. 3A and B). **Importantly, MCF-7 cells lack caspase-3 due to a CASP3 exon 3 deletion (23a,23b). Despite this, numerous papers report anti-caspase-3 antibody immunoreactivity in MCF-7, which may reflect caspase-7 cross-reactivity, consistent with compensatory effector caspase activation (23a-c)'**.
- Finally, the text in the legend for Fig. 3 should be revised to the following: 'Figure 3. Tanshinone I activates **effector caspase** pathway and decreases the Bcl-2/Bax ratio in breast cancer cells. Cells were treated with tanshinone I in a concentration-dependent manner for 4 and 6 h, as described in the Materials and methods section, and total proteins were extracted. The levels of Bcl-2, Bax and **cleaved caspase 3 immunoreactivity** were determined in MCF-7 (A) and MDA-MB-231 (B) cells by western blot analysis. These results were confirmed by three independent experiments'.

The authors are grateful to the Editor of *International Journal of Oncology* for allowing them this opportunity to publish this Corrigendum, and all the authors agree with its publication. Note that these revisions do not grossly affect either the results or the conclusions reported in this study; furthermore, the authors apologize to the readership for any inconvenience caused.

